# Real-World Observational Study on Vildagliptin With Insulin (VIL-INS) or Vildagliptin and Metformin With Insulin (VIL-MET-INS) Therapy in Indian Patients With Type 2 Diabetes Mellitus

**DOI:** 10.7759/cureus.47190

**Published:** 2023-10-17

**Authors:** P Panneerselvam, Dibakar Biswas, Hema Singh, K Dilip Kumar, P Ravi Kumar, Pramila Kalra, Santosh Revankar, Sona Warrier

**Affiliations:** 1 Diabetes and Endocrinology, Aruna Diabetes Centre, Chennai, IND; 2 Diabetes and Endocrinology, Dr D Biswas Clinic, Kolkata, IND; 3 Diabetes and Endocrinology, Dr Hema's Clinic, Jaipur, IND; 4 Diabetes and Endocrinology, Careful Diagnostic Centre, Kolkata, IND; 5 Diabetes and Endocrinology, R K Diabetes and Endocrinology Centre, Ranchi, IND; 6 Diabetes and Endocrinology, Pramila's Clinic, Bengaluru, IND; 7 Scientific Services, USV Private Limited, Mumbai, IND

**Keywords:** uncontrolled hyperglycemia, oral anti-diabetic agents, vildagliptin and metformin treatment, weight loss, tolerability, hba1c, diabetes

## Abstract

Background and objective: The therapeutic use of vildagliptin and insulin (VIL-INS) or vildagliptin and metformin in combination with insulin (VIL-MET-INS) in the Indian scenario has yet to be explored by generating real-world evidence. Therefore, the present study aimed to evaluate the demographic, clinical characteristics, and treatment patterns of patients with type 2 diabetes mellitus (T2DM) in Indian settings in the above context.

Methodology: This observational study conducted at 600 healthcare centers in India retrospectively analyzed data of adult patients with T2DM who had been treated with either vildagliptin with insulin or a combination of vildagliptin and metformin with insulin. Data were collected from medical records and analyzed by appropriate statistical tests.

Results: A total of 12,603 patients with T2DM were included with a mean age of 53.4 years of which 63.8% were males. The majority of patients (n=6511; 51.7%) received a combination of vildagliptin and metformin on top of insulin. A significantly high proportion of patients in the age group of 18-40 years received this treatment compared to patients who were initiated on insulin treatment after vildagliptin and metformin combination (11.6% vs. 9.7%; P<0.001). Of all the patients, 70.0% were able to achieve target glycemic control with either VIS-INS or VIL-MET-INS. After treatment with VIL-INS or VIL-MET-INS, the mean glycated hemoglobin (HbA1c) levels significantly decreased with a mean change of 1.46%. Out of all patients, 13.5% experienced weight changes during treatment, with 67.4% of them showing weight loss. A total of 68 patients reported hypoglycemic events and among them, 49 patients had mild hypoglycemic events. Physician global evaluation of efficacy and tolerability showed a majority of patients rated their experience as good to excellent (86.3% and 86.0%, respectively).

Conclusion: Both treatment regimens were effective in terms of reduced HbA1c to achieve glycemic control. Furthermore, it is well tolerated without an increase in the risk of hypoglycemia or weight gain. Hence, this therapy has favorable outcomes for T2DM management in Indian clinical settings.

## Introduction

The increasing burden of type 2 diabetes mellitus (T2DM) remains a major public health concern worldwide. An estimated global prevalence of T2DM is 7079 individuals per 100,000 by 2030 and India is one of the countries where the incidence is steadily increasing [1]. Due to a genetic predisposition, Indians as a race are at a higher risk of developing insulin resistance compared to the White population. This increased risk, combined with the upsurge in risk factors such as family history, rising obesity levels, insulin resistance, and lifestyle changes due to urbanization is contributing to the continued rise of T2DM prevalence in India [2,3].

The treatment of T2DM becomes difficult as the disease progresses gradually. In patients with T2DM, the capacity for endogenous insulin secretion is impaired and therefore there is a frequent need for the use of insulin treatment in clinical settings. However, many clinicians are hesitant to start or increase insulin treatment due to the high rates of hypoglycemic events associated with it [4-6]. Therefore, effective management in patients with T2DM treated with insulin therapy to achieve better glycemic control without causing side effects is of critical importance.

Dipeptidyl peptidase (DPP)-4 inhibitors are the frequently used new category of oral hypoglycemic agents for T2DM due to their ability to improve glycemic control without increasing the risk of hypoglycemia [7]. The American Diabetes Association (ADA) and the European Association for the Study of Diabetes (EASD) included this treatment regimen in the therapeutic algorithm for T2DM management [8]. Vildagliptin is a selective inhibitor of DPP-4, which works efficiently in enhancing glycemic control in patients with T2DM by increasing insulin secretion, decreasing glucagon secretion, slowing down gastric emptying, and enhancing pancreatic islet function [9]. The treatment regimen with vildagliptin along with insulin (VIL-INS) for 24 months was safe and effective in reducing glycated hemoglobin (HbA1c) levels, the dose and frequency of insulin injections, and the risk of hypoglycemia in patients with T2DM [7]. In Asian patients, vildagliptin as monotherapy or in combination with insulin or metformin showed remarkable improvements in HbA1c levels, with reduced risks of developing hypoglycemia [10,11]. Similar results were reported by the study of Kothny et al. [12], in which VIL-INS or a combination of vildagliptin and metformin with insulin (VIL-MET-INS) significantly reduced the HbA1c level with low risk of hypoglycemia and weight gain in patients with T2DM. Moreover, VIL-INS showed improvement in glycemic control with an associated insulin-sparing effect [9]. VIL-INS showed reduced HbA1c and frequency and dosage of insulin with a low risk of hypoglycemia [7]. Additionally, this combination was well tolerated in patients with T2DM with severe renal impairment providing a renoprotective effect [13].

Several studies have demonstrated the safety and tolerability of VIL-INS in patients with T2DM [7,11]. However, the therapeutic use of VIL-INS or VIL-MET-INS in the Indian scenario has yet to be explored by generating real-world evidence. Therefore, the present real-world study aimed to evaluate demographic data, clinical characteristics, and treatment patterns of VIL-INS or VIL-MET-INS therapy for patients with T2DM and comorbidities in Indian settings.

## Materials and methods

Study design

This real-world, retrospective, multicentric, observational analysis was conducted across 600 healthcare centers in India, utilizing medical records of adult patients with T2DM who had received treatment with VIL-INS or VIL-MET-INS. Data were collected retrospectively from the medical records of eligible patients, including demographic characteristics, duration of disease, co-morbidities, concomitant medications, and dosage patterns from selected clinics and hospitals.

The approvals for the retrospective observational analysis were obtained from the independent ethics committee, CLINICOM (approval number: O158O/O3.09.2019). The study was conducted in accordance with the Declaration of Helsinki and the International Council for Harmonization-Good Clinical Practice (ICH-GCP), as well as all applicable legislation regarding non-interventional studies.

Eligibility criteria

The study included patients of both sexes, aged 18 years or older, who had been treated with any dosage of VIL-INS or VIL-MET-INS for the treatment of T2DM. Patients with incomplete data or any condition deemed unsuitable for inclusion were excluded from the analysis.

Data collection

The outcome of this observational data analysis was evaluated by the number and percentage of patients in all age groups receiving VIL-INS or VIL-MET-INS, patients receiving insulin of various types and strengths, patients on oral antidiabetic drugs (OADs) along with VIL-INS or VIL-MET-INS, patients with reduction in daily insulin injections and dosage, duration of VIL-INS or VIL-MET-INS, concomitant conditions and therapies, and the current and previous HbA1c levels, hypoglycemic events, any adverse events, and weight change during the course of therapy.

Definitions

Improvement in HbA1c status was defined by HbA1c level < 7% (at follow-up) as compared to the baseline HbA1c status. Hypoglycemia was defined based on physicians' clinical evaluations, where they rely on their assessments and observations to report instances of low blood glucose levels (<70 mg/dL).

Statistical analysis

The data was analyzed using IBM SPSS Statistics for Windows, Version 23.0 (Released 2015; IBM Corp., Armonk, New York, United States). Descriptive statistics, including mean and standard deviation (SD) or median and interquartile range (IQR) were used to summarize demographic characteristics of the patients, while frequency and percentages were used for categorical variables. The chi-square test was used to compare qualitative variables between groups, while paired sample t-test was used to compare pre- and post-treatment HbA1c levels. p<0.05 was considered statistically significant.

## Results

**Figure 1:**
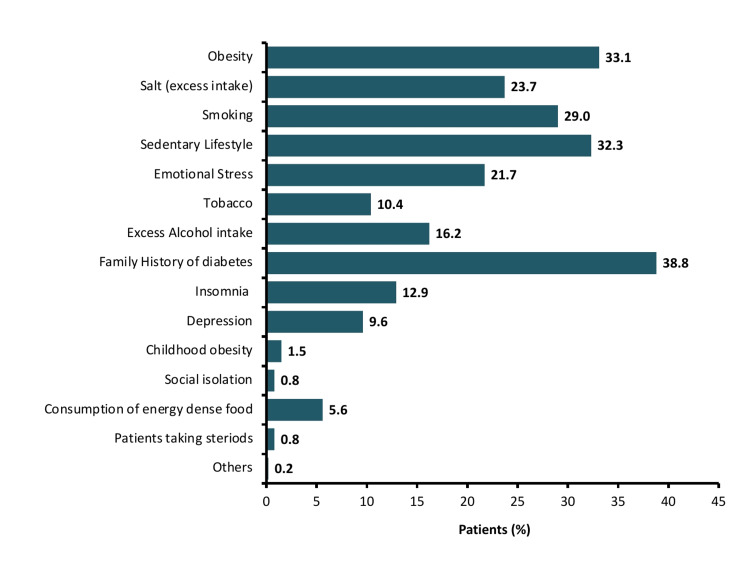
Risk factors associated with type 2 diabetes mellitus in the study population Others, risk factors included chronic obstructive pulmonary disease, polycystic ovary disease, amnesia, and gestational diabetes.

A total of 12,603 patients with T2DM were included in this observational analysis. The prevalence of males (63.8%) was higher than females (36.2%). The mean (SD) age of patients was 53.4 (10.5) years and the median duration of T2DM was 5.3 years. Other demographic details are summarized in Table 1. The common diabetic complications were neuropathy (23.9%) followed by depression (10.9%), retinopathy (10.7%), and nephropathy (10.2%). The majority of patients had dyslipidemia (42.5%), hypertension (27.5%), and coronary artery disease (CAD) (2.3) as comorbid conditions. A family history of diabetes (38.8%) was the most common risk factor associated with these patients (Figure 1).

**Table 1:** Demographic characteristics of study population ^#^N=12603 unless otherwise specified. *Others: diabetic complications included cheiroarthropathy, dental, hypothyroidism, osteoarthritis in both knee joints, soft tissue infections, recurrent perineal abscess, and weakness; **Others, comorbid conditions included hyperthyroidism, hypothyroidism, peptic ulcer disease, renal calculi disease, polycystic ovary disease. CAD, coronary artery disease; CKD, chronic kidney disease; T2DM, type 2 diabetes mellitus; HTN, hypertension, IQR, interquartile range; SD, standard deviation; T2DM, type 2 diabetes mellitus

Parameter	Patients (n=12,603)#
Sex	
Female	4561 (36.2)
Male	8042 (63.8)
Age (years), (n=12585), mean (SD)	53.4 (10.5)
Body mass index (kg/m2), mean (SD)	27.1 (4.3)
Area of stay	
Metropolitan	1586 (22.6)
Urban	4661 (37.0)
Semi-urban	4072 (32.3)
Rural	2284 (18.1)
Duration of T2DM (years), median (IQR)	5.3 (3.4-8.2)
Diabetic complications	
Neuropathy	3010 (23.9)
Depression	1379 (10.9)
Retinopathy	1345 (10.7)
Nephropathy	1280 (10.2)
Obstructive sleep apnoea	422 (3.3)
Osteoarthritis	408 (3.2)
Vaginal dryness in women	382 (3.0)
Erectile dysfunction	370 (2.9)
Others*	27 (0.2)
Comorbid conditions	
Dyslipidemia	4096 (42.5)
Hypertension	3468 (27.5)
CAD	291 (2.3)
CKD	26 (0.2)
Others**	23 (0.2)

In the age group 18-40 years, a significantly high proportion of patients received vildagliptin and metformin treatment on top of insulin compared to patients who were initiated on insulin treatment after vildagliptin and metformin combination (11.6% vs. 9.7%; P<0.001). The HbA1c level at the time of initiation of insulin was between 7.5-9% in 50.6% and >9% in 44.0% of the patients taking VIL-MET-INS, while HbA1c level >9% and 7.5-9% in 51.0% and 46.2%, respectively, who received vildagliptin and metformin combination on top of insulin (P<0.001). The majority of patients received (n=6511) vildagliptin and metformin combination on top of insulin, while other patients (n=6092) had insulin treatment initiation after vildagliptin and metformin combination therapy (Table 2).

**Table 2:** Pattern of treatment according to age group and HbA1c level Data shown as n (%). * n=6092; ** n=6511; unless otherwise specified HbA1c, glycated hemoglobin

Parameters	Insulin treatment initiation after vildagliptin and metformin combination (n=6092)*	Vildagliptin and metformin combination on top of Insulin (n=6511)**	P value
Age group (years)	(n=6084)	(n=6499)	
18-40	588 (9.7)	753 (11.6)	<0.001
41-60	4044 (66.5)	4341 (66.8)	
>60	1452 (23.8)	1405 (21.6)	
HbA1c level at initiation (%)			
<7.5	330 (5.4)	178 (2.7)	<0.001
7.5-9	3082 (50.6)	3010 (46.2)	
>9	2680 (44.0)	3323 (51.0)	

**Figure 2:**
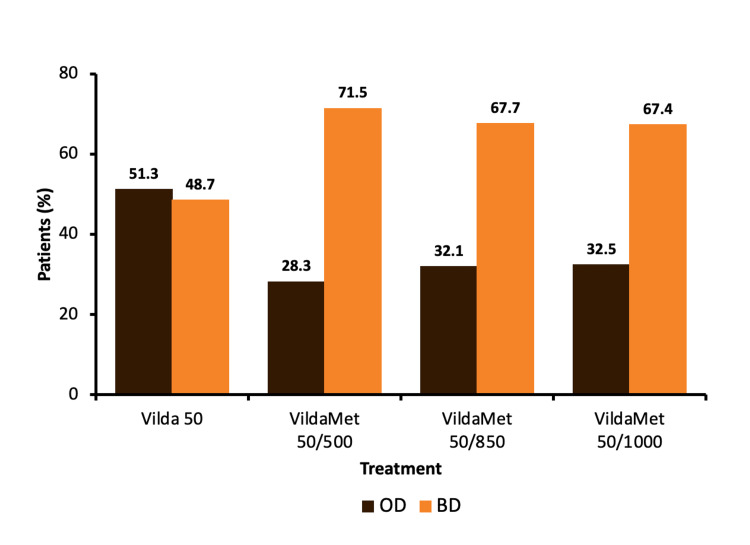
Vildagliptin and/or metformin dosage frequency

**Figure 3:**
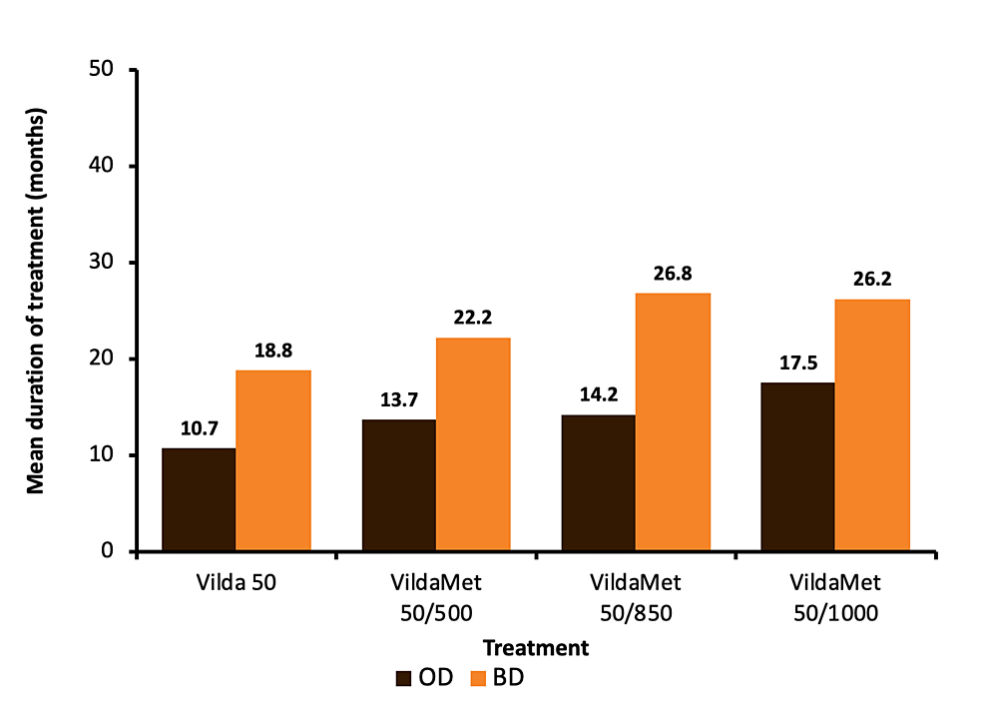
Vildagliptin and/or metformin duration of treatment

A treatment pattern of VIL-MET-INS is depicted in Table 3. Vildagliptin 50 mg with metformin 500 mg was the most commonly prescribed combination with insulin (63.3%). Twice daily was the most frequently used dosage pattern in the treatment of vildagliptin 50 mg plus metformin 500 mg (71.5%), vildagliptin 50 mg plus metformin 850 mg (67.7%), and vildagliptin 50 mg plus metformin 1000 mg (67.4%) (Figure 2). The mean duration of treatment in patients receiving a once-daily dose of VIL-MET-INS was in the range of 10.7 months to 17.5 months while in patients receiving a twice-daily treatment pattern the mean duration of treatment was in the range of 18.8 months to 26.8 months (Figure 3).

**Table 3:** Treatment patterns of VIL-INS and VIL-MET-INS Data shown as n (%). ^#^N=12603, unless otherwise specified. Any other* reasons for dosage up-titration include glucose control and PPG. Any other** reasons for dosage down-titration include added insulin, cost, intolerable to metformin dose, nausea, and dyspnea. Any other, patients who were on concomitant diabetic medication included meglitinides. Others, patients who were on concomitant non-diabetic medication included anti-inflammatory, antacid, anti-oxidants, diuretics, and vitamins. AGIs, alpha-glucosidase inhibitors; FPG, fasting plasma glucose; GLP1, glucagon-like peptide-1; HbA1c, glycated hemoglobin; PPG, post-prandial plasma glucose; SGLT2i, sodium-glucose co-transporter-2 inhibitor; VIL-MET-INS: vildagliptin and metformin with insulin; VIT-INS: vildagliptin with insulin

Parameters	Number of patients (N=12603)#
Treatment pattern	
Vildagliptin 50 mg	2270 (18.0)
Vildagliptin 50 mg + Metformin 500 mg	7975 (63.3)
Vildagliptin 50 mg + Metformin 850 mg	823 (6.5)
Vildagliptin 50 mg + Metformin 1000 mg	1535 (12.2)
Insulin treatment (n=9589)	
Long acting	3817 (39.8)
Fast acting	2261 (23.6)
Short acting	1416 (18.2)
Rapid acting	734 (7.7)
Ultra long-acting	322 (3.4)
Intermediate-acting	248 (2.6)
Others	457 (4.8)
Dose titration of vildagliptin or vildagliptin and metformin combination done during treatment	423 (3.4)
Dosage up-titration	358 (84.6)
Dosage down-titration	65 (15.4)
Reasons for dosage up-titration [n=358]	
Achieved HbA1c goal	338 (94.4)
Reduced insulin resistance	27 (7.5)
To lower the risk of hypoglycemia	2 (0.6)
Any other*	10 (2.8)
Reasons for dosage down-titration [n=65]	
Improved HbA1c level	21 (32.3)
Controlled FPG	4 (6.2)
Controlled PPG	2 (3.1)
To lower the risk of hypoglycemia	10 (15.4)
Due to kidney disease	20 (30.8)
Any other**	6 (9.2)
Dose titration of insulin done	1221 (9.7)
Dosage up-titration	502 (41.1)
Dosage down-titration	719 (58.9)
Concomitant anti-diabetic medication	2580 (20.5)
Sulfonylureas	1837 (71.2)
SGLT2i	431 (16.7)
AGIs	431 (16.7)
Thiazolidinedione	304 (11.8)
GLP1 agonist	22 (0.8)
Any other	53 (2.1)
Concomitant non-diabetic medications, [n=6196]	
Antihypertensive	3577 (57.7)
Antiplatelet	504 (8.1)
Statins	1774 (28.6)
Others	725 (11.7)

**Figure 4:**
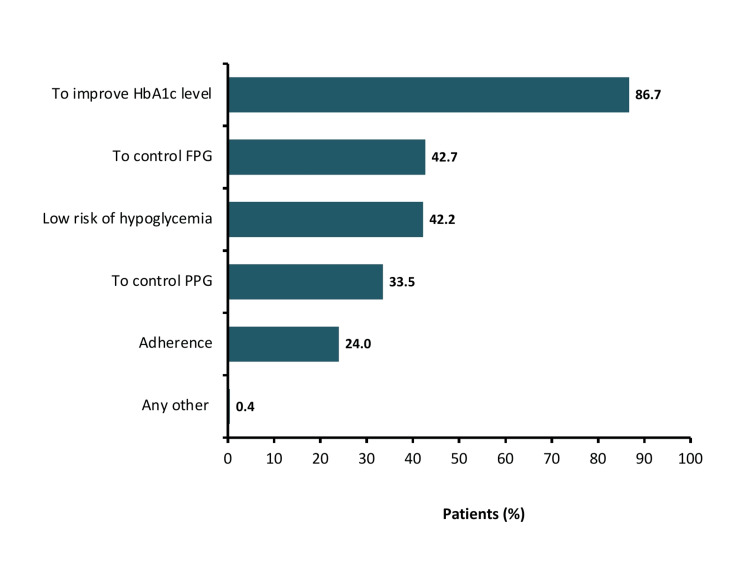
Reason for starting VIL-INS or VIL-MET-INS HbA1c, glycated hemoglobin; FPG, fasting plasma glucose; PPG, postprandial plasma glucose; VIL-INS: vildagliptin with insulin; VIL-MET-INS: vildagliptin and metformin with insulin Any other reason included foot ulcers, tooth extraction, onset of renal failure, to preserve beta cell function, weight neutral, reduce insulin dose.

Among insulin, Glargine (27.1%) was the most commonly prescribed insulin therapy, followed by human mixtard (22.7%) and human insulin (12.9%). The most common reasons for starting VIL-INS or VIL-MET-INS were to improve HbA1c level (86.7%), to control FPG (42.7%), and low the risk of hypoglycemia (42.2%) (Figure 4). A total of 423 (3.4%) patients required dose titration of VIL-INS or VIL-MET-INS during the treatment and out of these, 358 patients required dosage up-titration (Table 3). The most common reason given for dosage up-titration was to achieve the glycemic goal (72.6%). However, down-titration was required in 65 patients and improved HbA1c level (n=21) and due to kidney disease (n=20) were the most common reasons. There were 502 patients who required an up-titration of and 719 who needed a down-titration of insulin. The use of concomitant anti-diabetic medication was observed in only 20.5% of patients, where sulfonylurea (n=1837) was the most commonly prescribed. In concomitant non-diabetic medications, the most common class of drugs was antihypertensive (57.7%), followed by statins (28.6%), and antiplatelet (8.1%).

**Figure 5:**
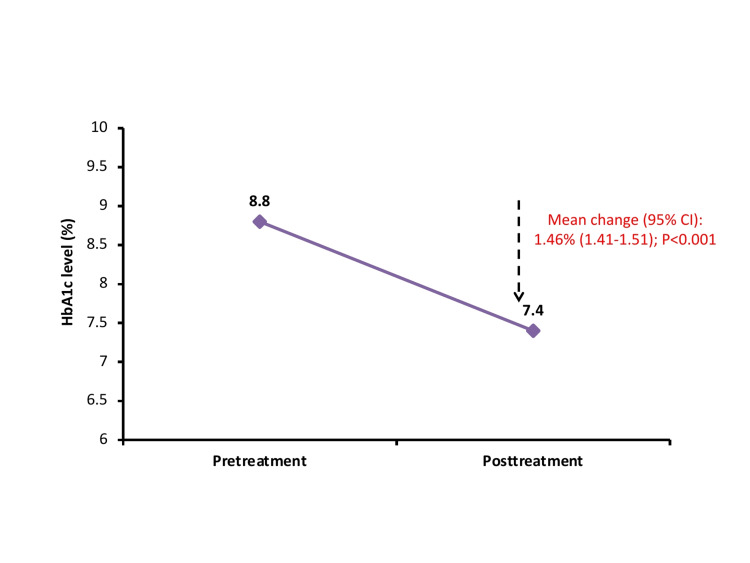
Mean change in HbA1c levels from pre-treatment to post-treatment HbA1c, glycated hemoglobin

Around 70.0% of patients achieved the target level of blood glucose control when treated with VIL-INS or VIL-MET-INS (Table 4). The mean HbA1c levels were significantly reduced after the treatment (from 8.8% to 7.4%) with VIL-INS or VIL-MET-INS with a mean change of 1.46% (95% CI, 1.41-1.51; P<0.001) (Figure 5). During the treatment, 13.5% of patients experienced changes in weight, with 67.4% of patients experiencing weight loss and 32.6% showing weight gain. Most patients experienced weight reduction (n=779) or elevation (n=394) up to 2 kgs. A total of 68 patients reported hypoglycemic events, with 49 of them experiencing mild events, as indicated in Table 4. The majority of patients (86.3% and 86.0%) were rated good to excellent in terms of efficacy and tolerability by physicians’ global evaluations.

**Table 4:** Observations related to glycemic control and weight alterations Data shown as n (%), unless otherwise specified. *N=12603, unless otherwise specified HbA1c, glycated hemoglobin; IQR, interquartile range

Parameters	Number of patients (N=12603)*
Patients with glycemic goal achieved	8827 (70.0)
Glycemic values (%), median (IQR)	
HbA1c at last 12 months [n=191]	8.0 (8.0-9.0)
HbA1c at last 9 months [n=202]	8.0 (7.0-9.0)
HbA1c at last 6 months [n=302]	8.0 (7.0-9.0)
HbA1c at last 3 months [n=299]	7.0 (7.0-8.0)
HbA1c at last 1 month [n=459]	7.0 (7.0-8.0)
Patients with weight changes during the therapy	1700 (13.5)
a) Decreased weight (kg)	1146 (67.4)
0-2	779 (67.8)
2-4	293 (25.6)
>4	74 (6.6)
b) Increased weight (kg)	554 (32.6)
0-2	394 (71.1)
2-4	131 (23.6)
>4	29 (5.3)
Lipid parameters (mg/dL), median (IQR)	
Triglycerides [n=1028]	162.0 (145.0-200.0)
Total cholesterol [n=1139]	205.0 (170.0-230.0)
Low density lipoprotein [n=1069]	130.0 (95.0-150.0)
High density lipoprotein [n=1034]	42.0 (38.0-48.0)
Adverse events reported	[n=68]
Mild hypoglycemia	49 (72.1)
Dizziness	3 (4.4)
Loss of memory	1 (1.5)
Hypoglycemia	3 (4.5)
Palpitations and sweating	2 (3.0)
Sweating and anger	1 (1.5)
Urinary tract infection	2 (3.0)
Swelling	2 (3.0)
Itching	1 (1.5)
Physician global evaluation of efficacy	
Excellent	1813 (14.4)
Very good	3716 (29.5)
Good	5340 (42.4)
Average	1425 (11.3)
Fair	309 (2.4)
Physician global evaluation of tolerability	
Excellent	1853 (14.7)
Very good	3553 (28.2)
Good	5432 (43.1)
Average	1395 (11.1)
Fair	370 (2.9)

The patient-centric program was initiated in 36.7% of patients (Table 5). Among patient-centric programs, lifestyle modification (83.4%) was the most common, followed by a balanced diet plan (39.7%), and 30-min walk (38.8%). The moderate exercise program was reported in the majority of patients (64.6%). Around 80% of patients reported knowledge of diabetes disease on a good to excellent scale. Telephonic platform (81.5%) was the most commonly used platform for sharing knowledge on diabetes.

**Table 5:** Patients-centric program Data shown as n (%). *N=12603, unless otherwise specified. Other, prevention program included, leave smoking and tobacco chewing. Any other, diabetic knowledge-sharing platform included booklets, brochures, counseling, counseling by diabetes nurse.

Parameters	Number of patients (N=12603)*
Patients-centric program	
Yes	4627 (36.7)
No	7976 (63.3)
Patient-centric program, if yes	
Lifestyle medication	3861 (83.4)
Prevention of obesity	956 (10.7)
Home blood pressure monitoring	889 (19.2)
Home glucose monitoring	1395 (30.1)
Family support	648 (14.0)
Frequent lab parameter tested	413 (8.9)
Low sodium diet	722 (15.6)
Balanced diet plan	1836 (39.7)
30 minute walk	1793 (38.8)
Regular follow-up with physician	1025 (22.2)
Glycemic control	898 (19.4)
Blood pressure control	518 (11.2)
Timely screening for long term complication	430 (9.3)
Psychological and social support	234 (5.1)
Others	2 (0.01)
Exercise program	
Regular	3024 (24.0)
Moderate	8141 (64.6)
No physical activity	1438 (11.4)
Knowledge on diabetes disease	
Excellent	1132 (9.0)
Very good	4507 (35.8)
Good	4472 (35.5)
Average	1808 (14.3)
Fair	684 (5.4)
Preferred diabetes knowledge sharing platform	
Digital	1818 (14.4)
If digital (n=1794)	
Online	1031 (57.5)
Offline	763 (42.5)
Telephonic	10266 (81.5)
Any other	519 (4.1)
Patient adherence to medication	
Forget medication once a week	1363 (10.8)
Forget medication once a month	1961 (15.6)
Forget medication once a year	1505 (11.9)
Partial compliance for medication	2905 (23.1)
Compliance for medication	4869 (38.6)

## Discussion

The combination of oral anti-diabetic agents with insulin can lower glycemic index and improve clinical outcomes, reducing diabetes-related health complications. The present study evaluated VIL-INS or VIL-MET-INS to manage T2DM in patients with uncontrolled hyperglycemia. Moreover, associated conditions and therapies, treatment patterns, glycemic control, hypoglycemic events, impact on weight and patient-centric program were evaluated. 

The key findings of this study showed that the majority of patients received vildagliptin or vildagliptin and metformin combination on top of insulin and a significantly higher proportion of patients between 18 and ≤40 years age group received this combination compared to insulin treatment initiation after vildagliptin and metformin combination (11.6% vs. 9.7%; P<0.001). Recent findings reported that the addition of vildagliptin to insulin therapy led to a substantial improvement in glycemic control [14]. Importantly, this improvement was achieved without an increased risk of hypoglycemia (low blood sugar levels) or weight gain. These results highlight the potential benefits of combining VIL-INS in terms of achieving better blood glucose management while minimizing the common adverse effects associated with intensive insulin therapy.

The concomitant use of insulin and a DPP-4 inhibitor was recommended by a joint position statement of the American Diabetes Association (ADA) and the European Association for the Study of Diabetes (EASD) in the therapeutic algorithm for T2DM management [8]. Evidence from clinical studies has established that vildagliptin reduces HbA1C in patients with T2DM who were poorly controlled with high doses of insulin [7,11,13-15]. In a recent 24-week, placebo-controlled study, vildagliptin 50 mg significantly reduced HbA1c in 449 patients with T2DM who were inadequately controlled by basal or premixed insulin (about 40 U/day) with or without metformin [12]. This was in accordance with the present study, where 12603 patients with T2DM received VIL-INS or VIL-MET-INS treatment to achieve target HbA1c. Of these, 6511 patients received vildagliptin or vildagliptin and metformin combination on top of insulin, and the remaining patients had insulin treatment initiation after vildagliptin or vildagliptin and metformin combination therapy.

A previous study reported vildagliptin 50 mg and metformin 500 mg were commonly prescribed for the management of T2DM [16]. In line with this, the present study observed that a combination of vildagliptin 50 mg and metformin 500 mg was the most common treatment pattern. Additionally, the majority of patients received this combination on top of insulin. In a previous study, vildagliptin 50 mg was compared with a placebo add-on to insulin and metformin therapy. This study indicated that vildagliptin significantly reduced HbA1c with or without metformin therapy (0.85%). Furthermore, in subgroups by ethnicity, HbA1c significantly reduced from baseline with 0.99% in Indian patients with T2DM [14]. In the current study, HbA1c reduction was in accordance with the previous studies [11,13,14] and greater than Kothny et al. [12].

Diabetes and coexisting conditions like hypertension and dyslipidemia are commonly reported in population-based studies conducted in India [17,18]. Similarly, the present study reported dyslipidemia (42.5%) and hypertension (27.5%) as the most common comorbid conditions in patients with T2DM.

The GALATA study assessed the safety and tolerability of vildagliptin and metformin treatment, monitoring common side effects of oral anti-diabetic agents, including hypoglycemia and gastrointestinal events, were monitored in this study [19]. The tolerability/safety profile of VIL-MET-INS was reported in the previous studies [11-14], indicating vildagliptin causes fewer hypoglycemic events with neutral body weight. Our finding reported that only 0.5% of patients experienced hypoglycemia during the course of the study. Though insulin is associated with the risk of hypoglycemia and weight gain, the addition of vildagliptin or vildagliptin and metformin improved glycemic control with an associated insulin-sparing effect in patients with T2DM. Weight reduction in the present study population agreed with previously reported favorable effects of vildagliptin add-on to insulin treatment [19].

Indian clinicians are very familiar with using VIL-INS or VIL-MET-INS. The majority of patients were rated physician global evaluation on a good to excellent scale in terms of efficacy and tolerability (86.3% and 86.0%).

This study was conducted in a real-world setting to demonstrate the clinical outcomes of using different doses of VIL-INS or VIL-MET-INS in patients with high HbA1c levels. Additionally, the combination was administered to patients with coexisting conditions and complications. This treatment was well tolerated with very few hypoglycemic events and weight loss, indicating a good safety and efficacy profile with this treatment.

Limitations

Despite the inclusion of a large number of patients in this study, its retrospective design poses a limitation. Furthermore, the study only included T2DM cases from healthcare centers in India, which may limit the generalizability of the findings to other populations. The study also did not record fasting and postprandial blood glucose levels. The analysis strength of the study parameters was further limited by the absence of data from patients who failed to report.

## Conclusions

VIL-INS or VIL-MET-INS proved to be an effective treatment for diabetes, successfully achieving the glycemic targets through lowered HbA1c levels. Notably, this treatment showed a low incidence of hypoglycemia and weight loss among Indian patients with comorbid conditions. Furthermore, no events of severe hypoglycemia were reported, indicating the favorable outcomes of this therapy in effectively managing T2DM in Indian settings with comorbidities.
